# Supplementary material for: Inclusive, exclusive and hierarchical atlas of NFATc1+/PDGFR-α+ cells in dental and periodontal mesenchyme
Source: eLife. 2024 Dec 20;13:RP100173. doi: 10.7554/eLife.100173 (PMC11661793; doi:10.7554/eLife.100173)
Supplement: Supplementary file 1. [file elife-100173-supp1.pdf]

**Supplemental data**

# Inclusive, Exclusive and Hierarchical Atlas of NFATc1$^+$/PDGFR-α$^+$ Cells in Dental and Periodontal Mesenchyme

Xue Yang[1, 2, *], Chuyi Han[1, *], Changhao Yu[1, 2], Bin Zhou[3], Ling Ye[1, 2, #], Feifei Li[1, 4, #], Fanyuan Yu[1, 2, #]

1 State Key Laboratory of Oral Diseases & National Clinical Research Center for Oral Diseases, West China Hospital of Stomatology, Sichuan University, China
2 Department of Endodontics, West China Hospital of Stomatology, Sichuan University, China
3 Center for Excellence in Molecular Cell Science, University of Chinese Academy of Sciences, Chinese Academy of Sciences, China
4 Department of Pediatric Dentistry, West China Hospital of Stomatology, Sichuan University, China
* These authors contribute equally.
# Corresponding Author. E-mail: yeling@scu.edu.cn; feidepingfanshijie@163.com; fanyuan_yu@outlook.com

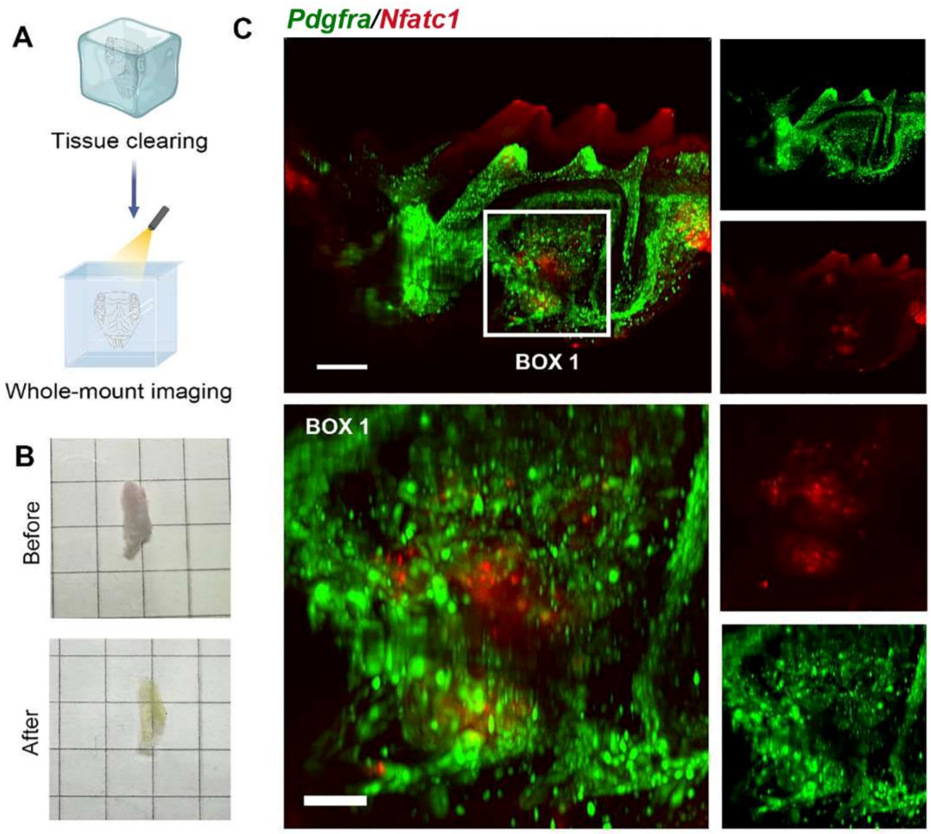


**Figure 2—figure supplement 1**. A. The tissue-clearing (TC) and whole-mount imaging procedure of mice maxilla. B. The images before & after the TC procedure of mice maxilla. C. The distribution of PDGFR-a+ and NFATc1+ cells from the section of XZ axis after 3D reconstruction of TC imaging. The image was from *Pdgfrα*$^{CreER}$ × *Nfatc1*$^{DreER}$ × LGRT mice (pulse). Box 1: alveolar bone. Scale bar = 300 μm for top row, 100 μm for bottom row.

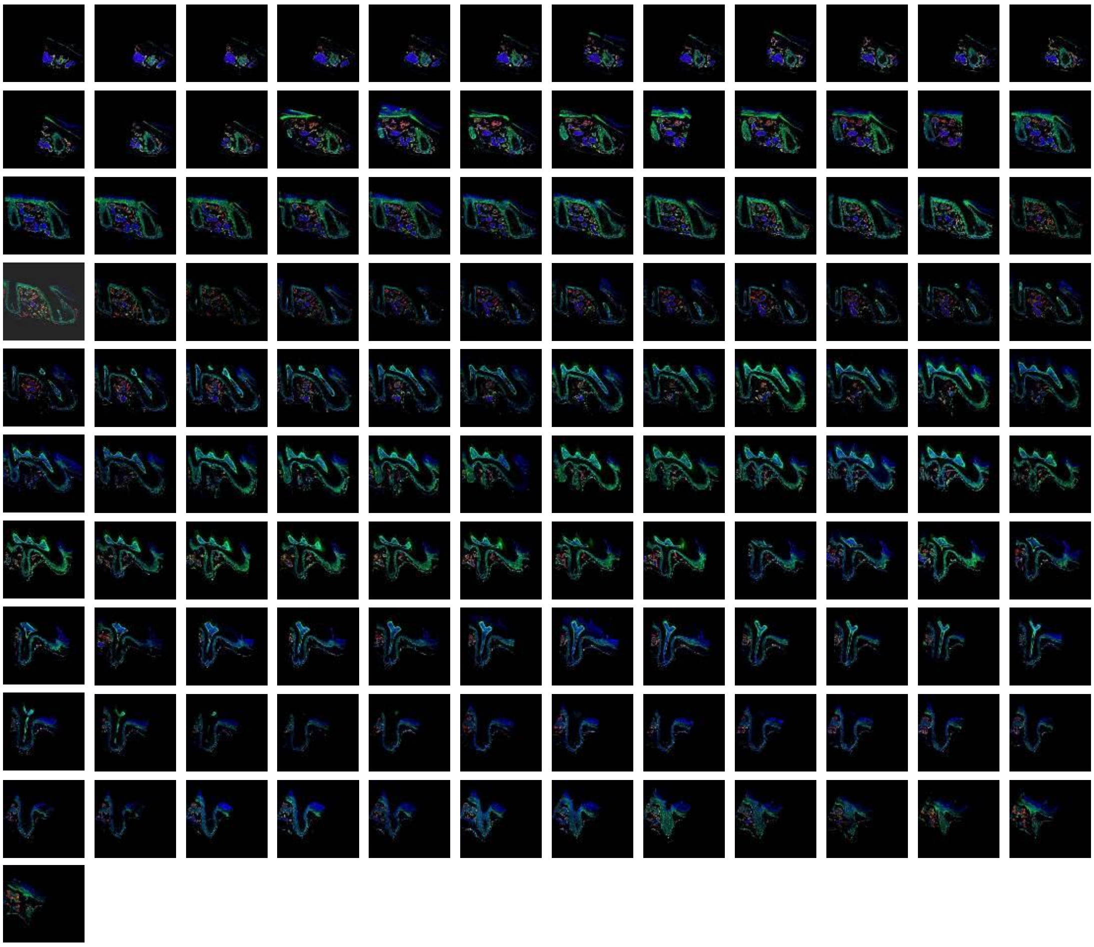

**Figure 3—figure supplement 1**. The total 121 slices of maxilla M1 of *Pdgfrα*$^{CreER}$ × *Nfatc1*$^{DreER}$ × LGRT mice sample (pulse). The images were acquired by confocal microscope, ZsGreen$^{+}$ cells in green, tdTomato$^{+}$ cells in red, DAPI in blue.

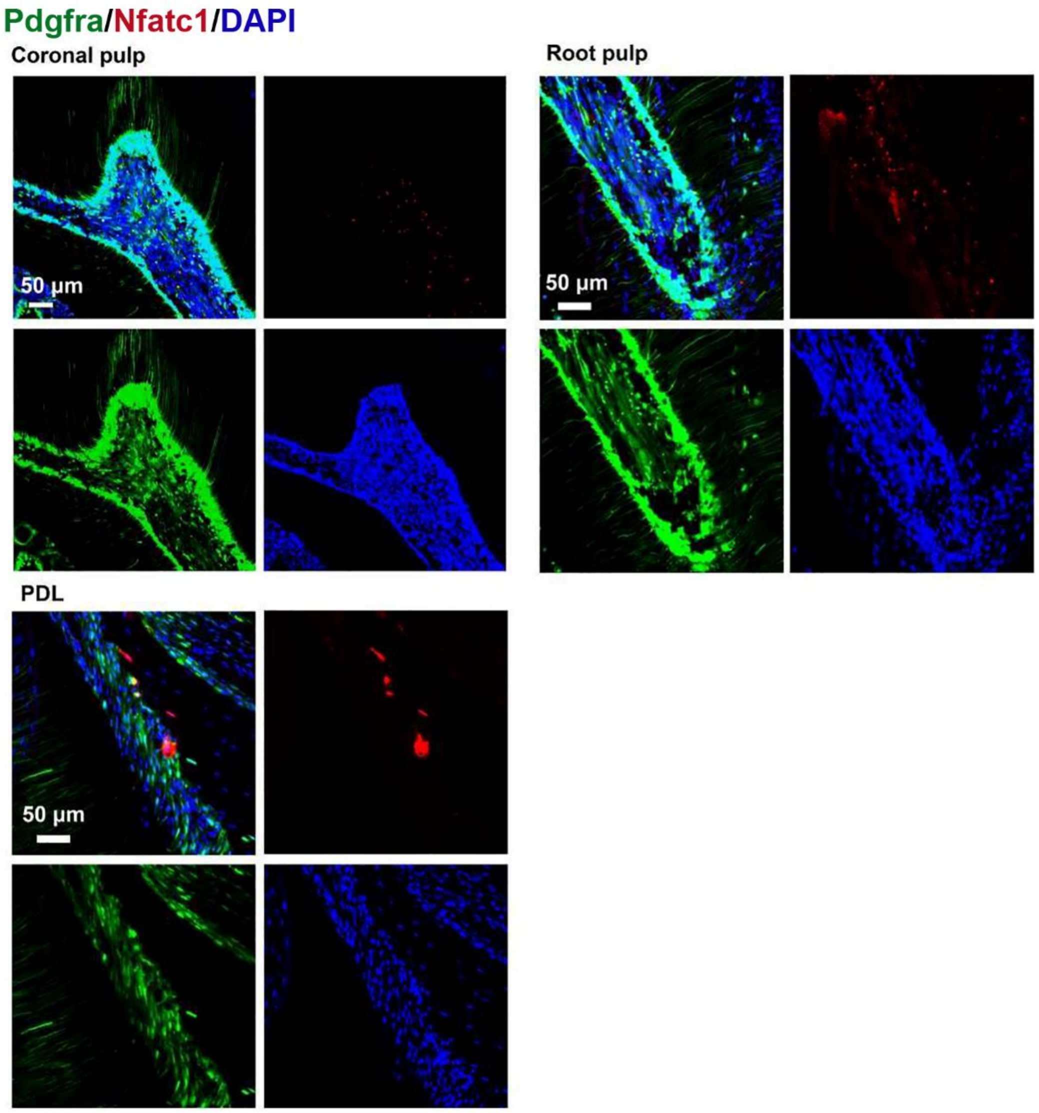


**Figure 3—figure supplement 2**. Representative images of coronal pulp, root pulp, and PDL of maxilla M1 of *Pdgfrα*$^{CreER}$ × *Nfatc1*$^{DreER}$ × LGRT mice sample (pulse). The images were acquired by confocal microscope.

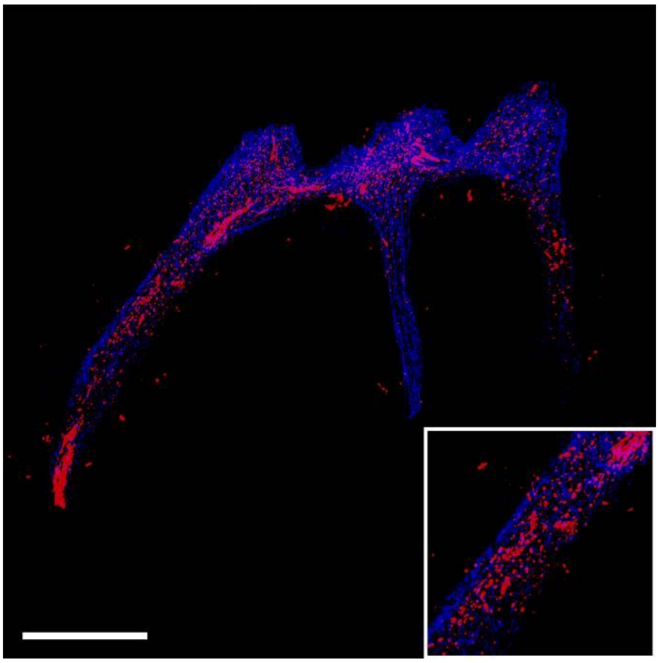

**Figure 3—figure supplement 3**. The tdTomato signal in pulp reconstructed by traditional serial section-based confocal imaging method (scale bar = 300 μm). The sample was from *Pdgfrα*$^{CreER}$ × *Nfatc1*$^{DreER}$ × LGRT mice (pulse).

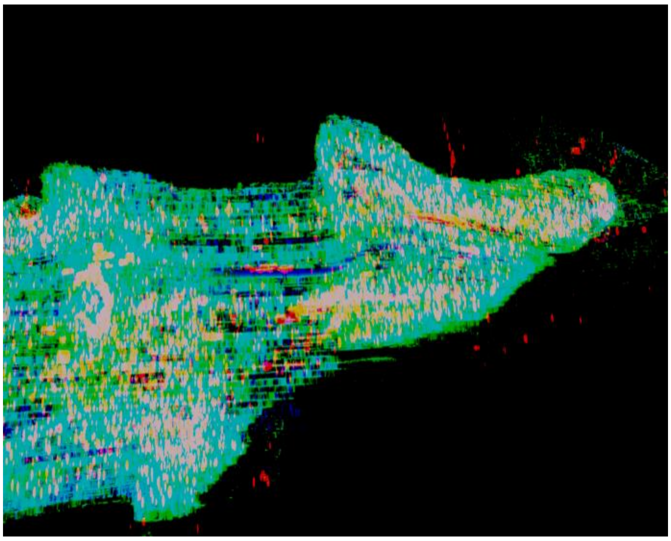

**Figure 3—figure supplement 4**. The discontinuities in the z-axis due to stratification of slices. The image was reconstructed by serial sections of maxilla M1 of *Pdgfrα*$^{CreER}$ × *Nfatc1*$^{DreER}$ × LGRT mice sample (pulse).

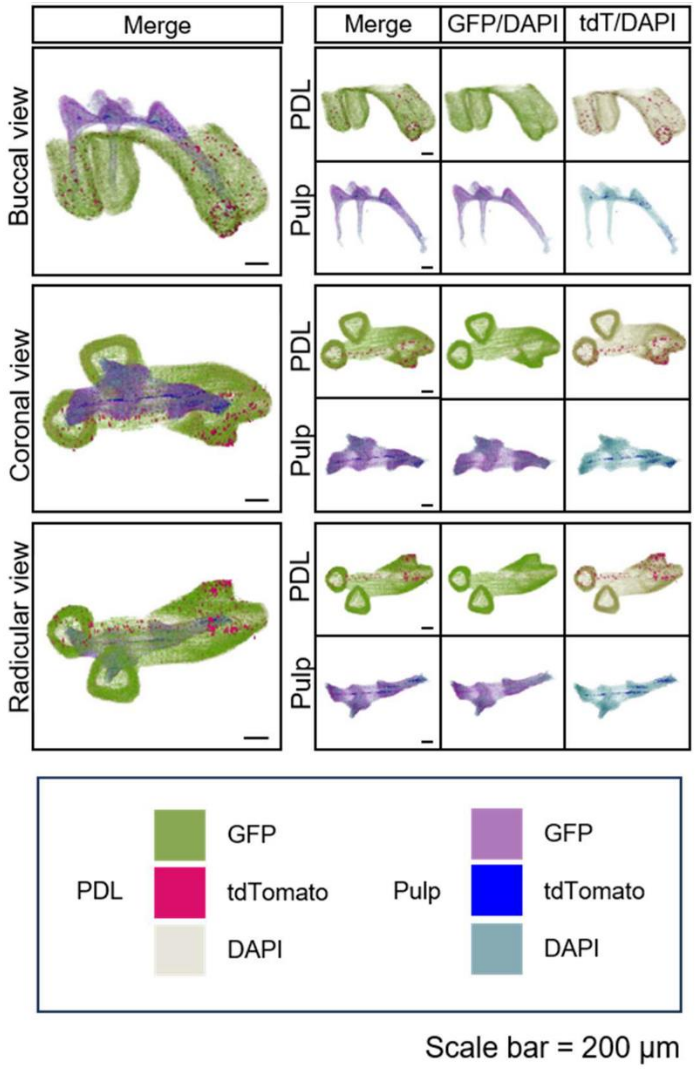


**Figure 3—figure supplement 5**. 3D reconstruction of maxilla M1 of *Pdgfrα*$^{CreER}$ × *Nfatc1*$^{DreER}$ × LGRT mice (pulse) by DICOM-3D; in PDL: ZsGreen$^{+}$ cells in green, tdTomato$^{+}$ cells in rose red; in pulp: ZsGreen$^{+}$ cells in purple, tdTomato$^{+}$ cells in blue. The image stack was also displayed in buccal view, coronal view, and radicular view of pulp and PDL, respectively.

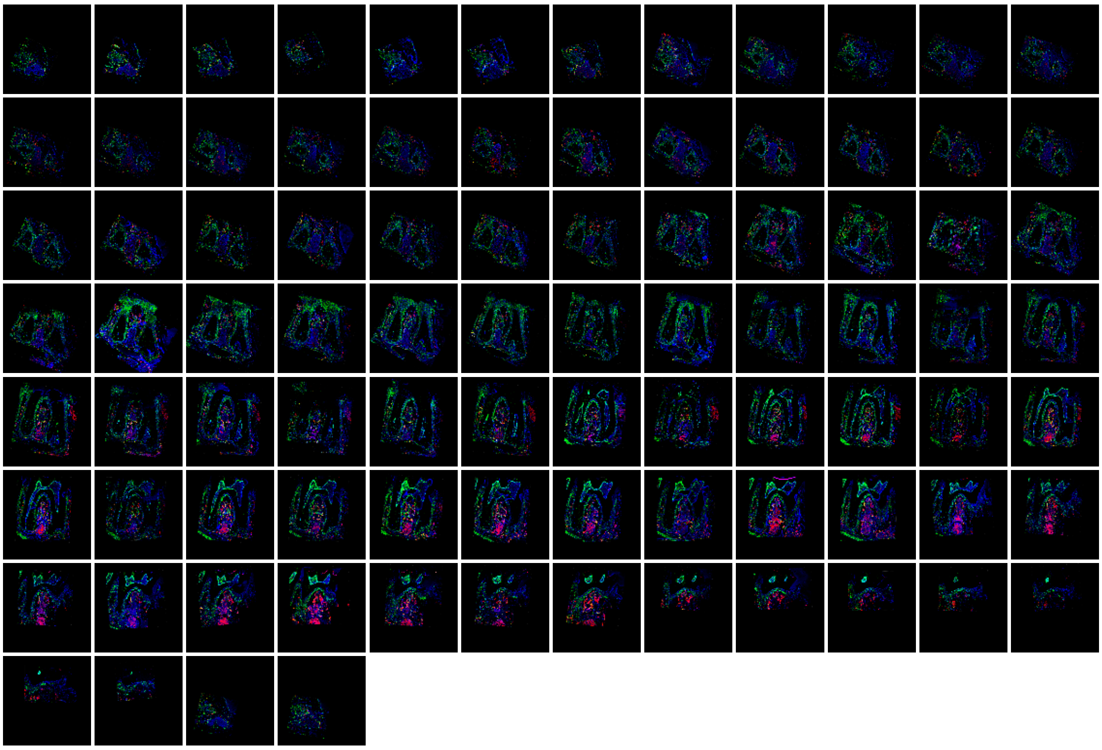

**Figure 4—figure supplement 1**. The total 88 slices of mandible M1 of *Pdgfrα*$^{CreER}$ × *Nfatc1*$^{DreER}$ × LGRT mice sample (pulse). The images were acquired by confocal microscope, ZsGreen$^{+}$ cells in green, tdTomato$^{+}$ cells in red, DAPI in blue.

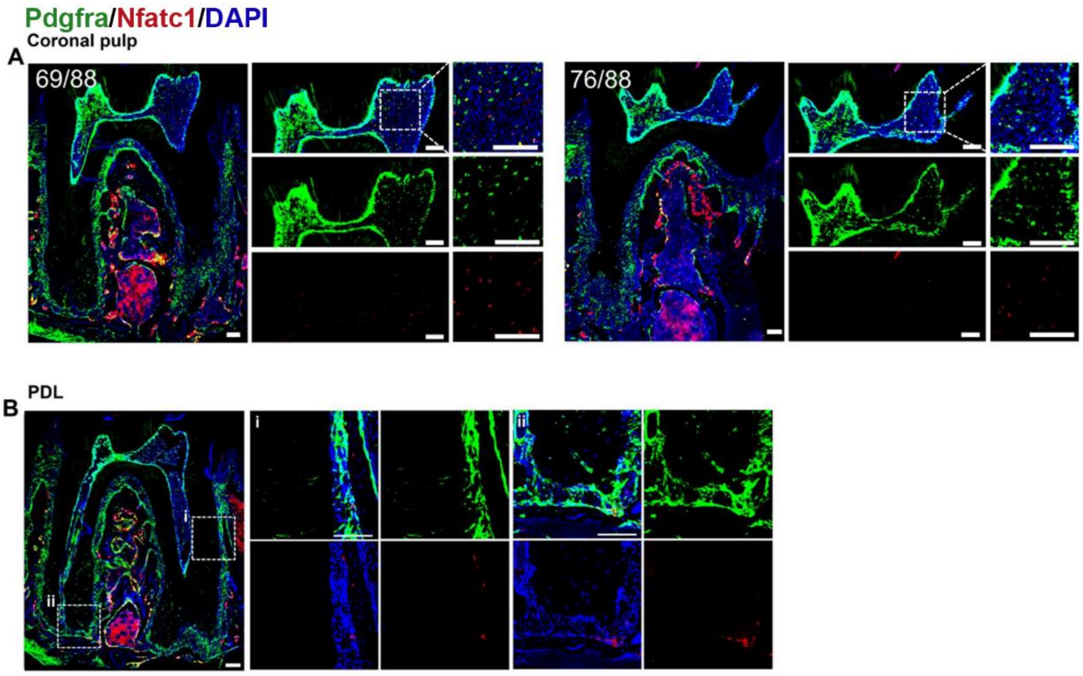


**Figure 4—figure supplement 2**. Representative images of coronal pulp (A) and PDL (B) acquired by confocal microscope of mandible M1 of *Pdgfrα*$^{CreER}$ × *Nfatc1*$^{DreER}$ × LGRT mice sample (pulse). (Scale bar = 100 μm).

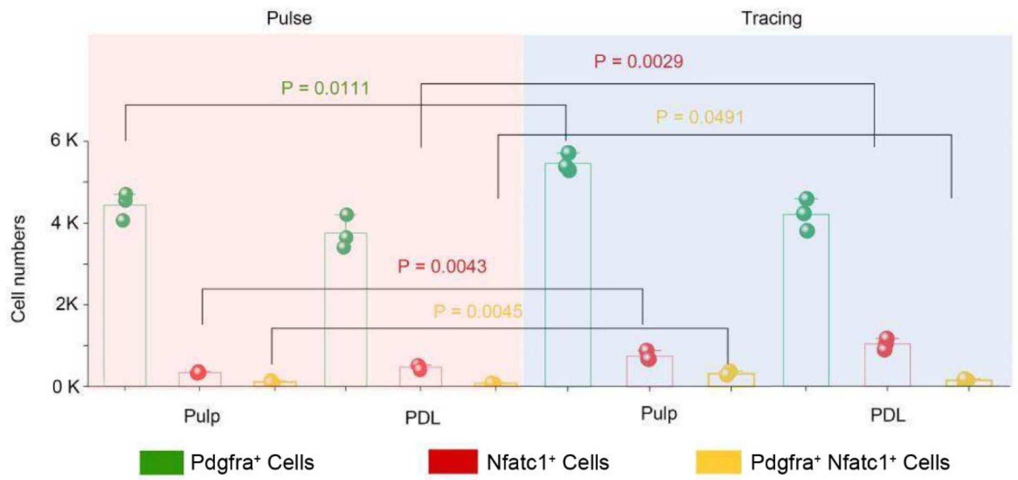


**Figure 5—figure supplement 1**. The number of PDGFR-α$^{+}$ cells, NFATc1$^{+}$ cells, and PDGFR-α$^{+}$&NFATc1$^{+}$ cells in pulp and PDL, respectively. The quantification was done on a z stack of images in Imaris using the automatic spot detection feature.

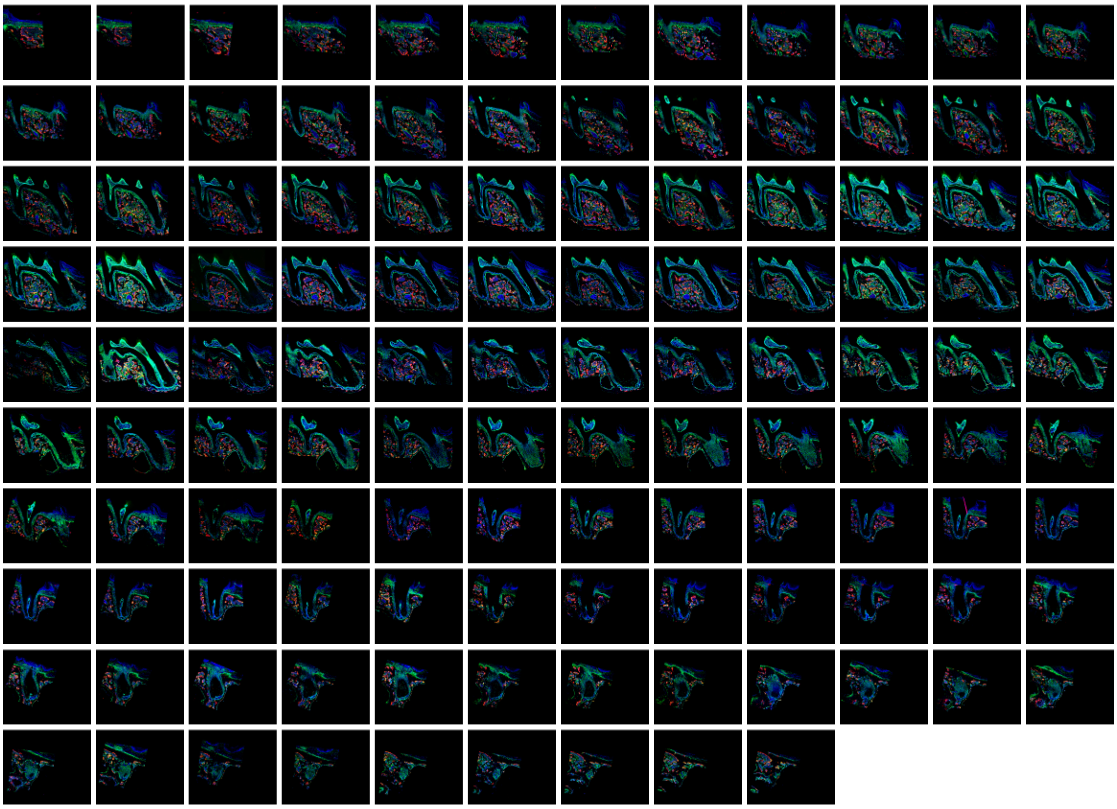

**Figure 6—figure supplement 1**. The total 117 slices of maxilla M1 of *Pdgfrα*$^{CreER}$ × *Nfatc1*$^{DreER}$ × LGRT mice sample (tracing). The images were acquired by confocal microscope, ZsGreen$^{+}$ cells in green, tdTomato$^{+}$ cells in red, DAPI in blue.

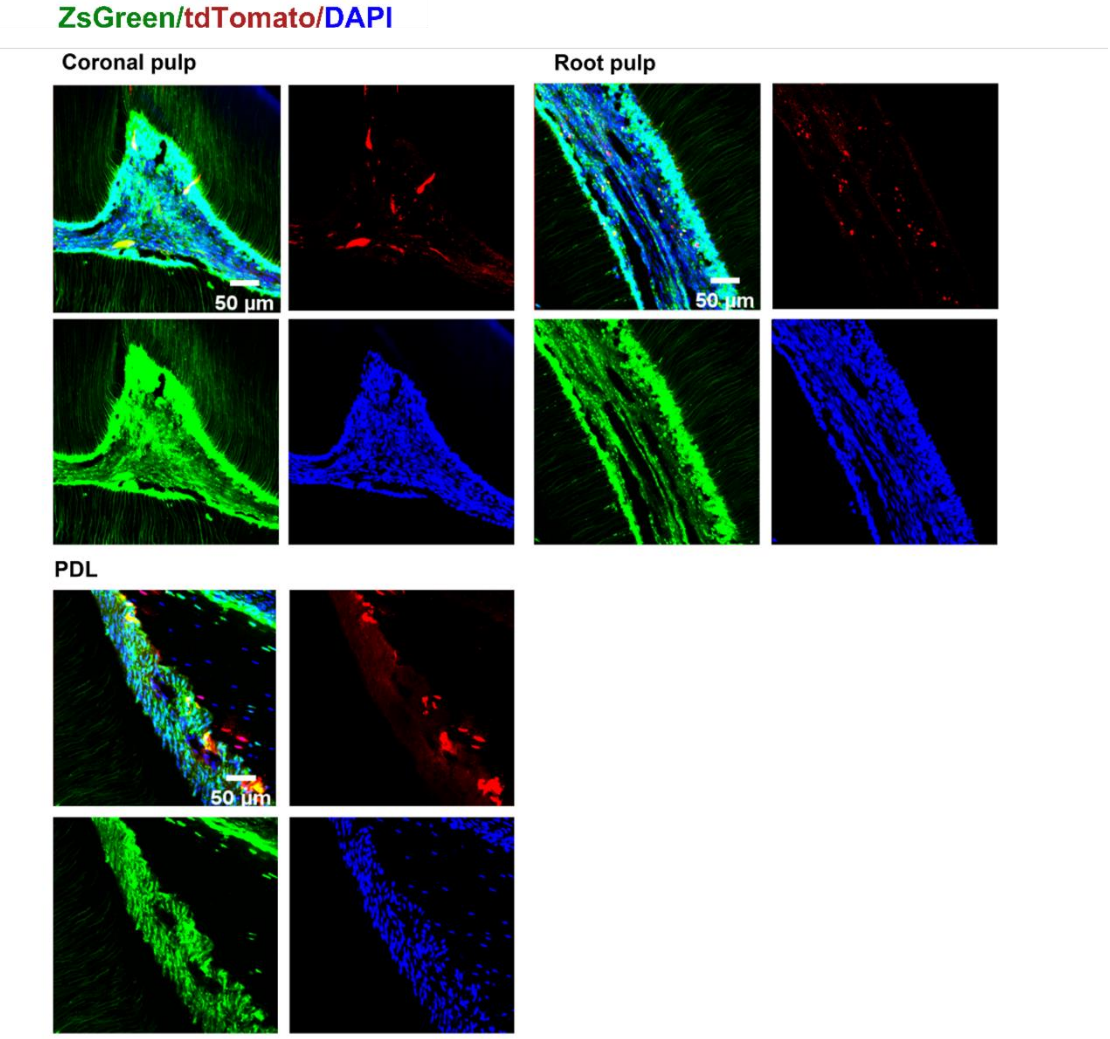


**Figure 6—figure supplement 2**. Representative images of coronal pulp, root pulp, and PDL of maxilla M1 of *Pdgfrα*$^{CreER}$ × *Nfatc1*$^{DreER}$ × LGRT mice sample (tracing). The images were acquired by confocal microscope (scale bar = 50 μm).

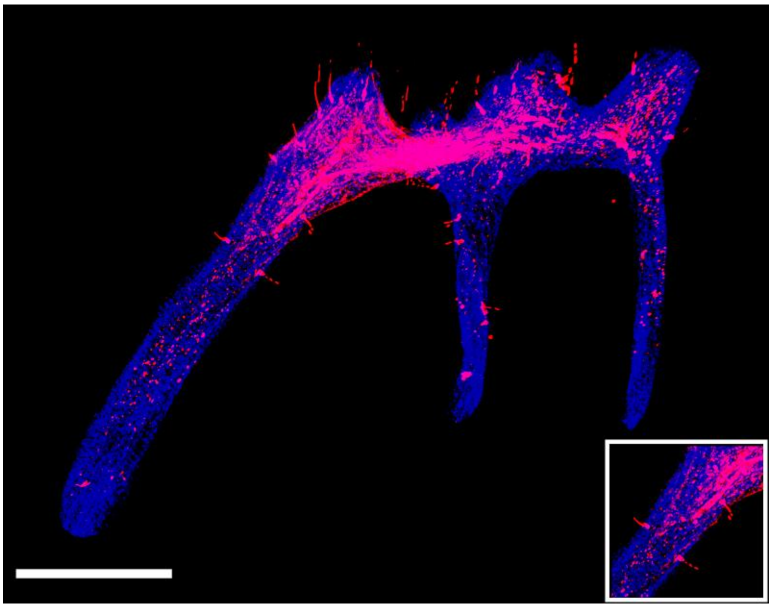

**Figure 6—figure supplement 3**. The tdTomato signal in pulp of maxilla M1 of *Pdgfrα*$^{CreER}$ × *Nfatc1*$^{DreER}$ × LGRT mice sample (tracing) reconstructed by traditional serial section-based confocal imaging method (scale bar = 300 μm).

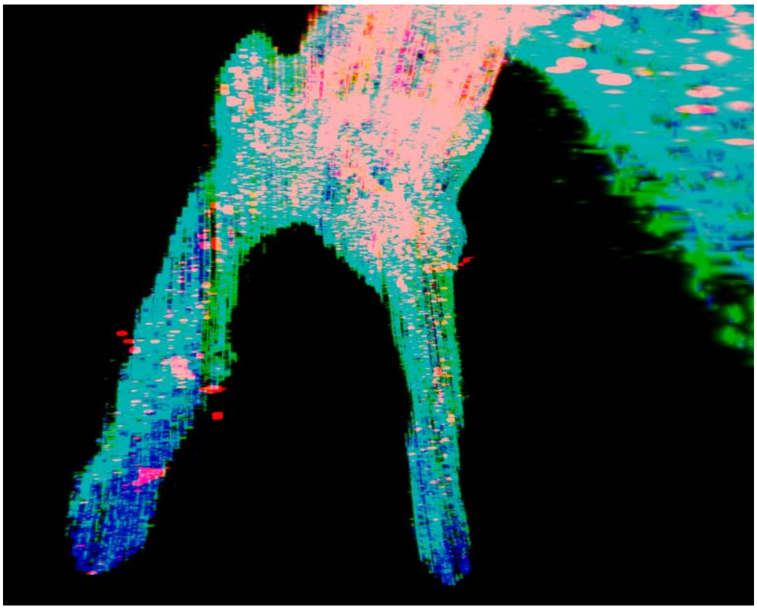

**Figure 6—figure supplement 4**. The discontinuities in the z-axis due to stratification of slices. The image was reconstructed by serial sections of maxilla M1 of *Pdgfrα*$^{CreER}$ × *Nfatc1*$^{DreER}$ × LGRT mice sample (tracing) using Imaris. ZsGreen$^{+}$ cells in green, tdTomato$^{+}$ cells in red, DAPI in blue.

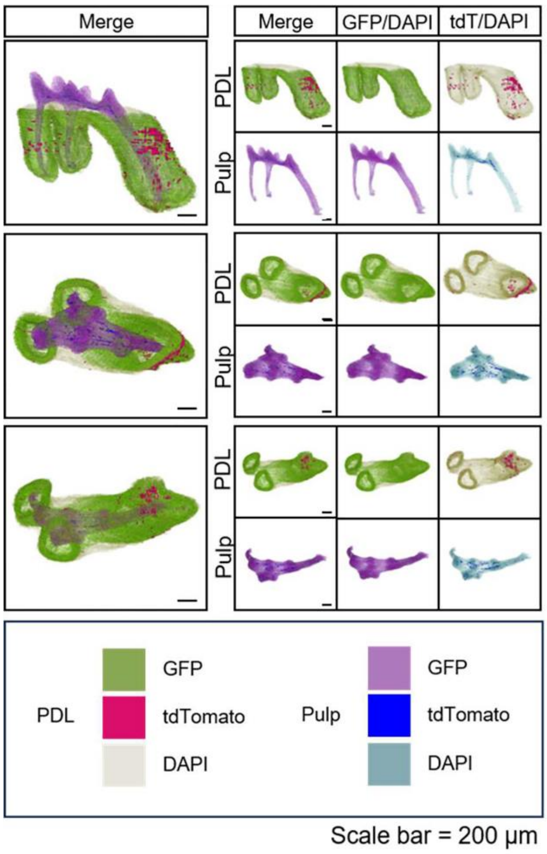


**Figure 6—figure supplement 5**. 3D reconstruction of maxilla M1 of *Pdgfrα*$^{CreER}$ ×

*Nfatc1*$^{DreER}$ × LGRT mice (tracing) by DICOM-3D; PDL: ZsGreen$^{+}$ cells in green, tdTomato$^{+}$ cells in rose red; pulp: ZsGreen$^{+}$ cells in purple, tdTomato$^{+}$ cells in blue. The image stack was also displayed in buccal view, coronal view, and radicular view of pulp and PDL, respectively.

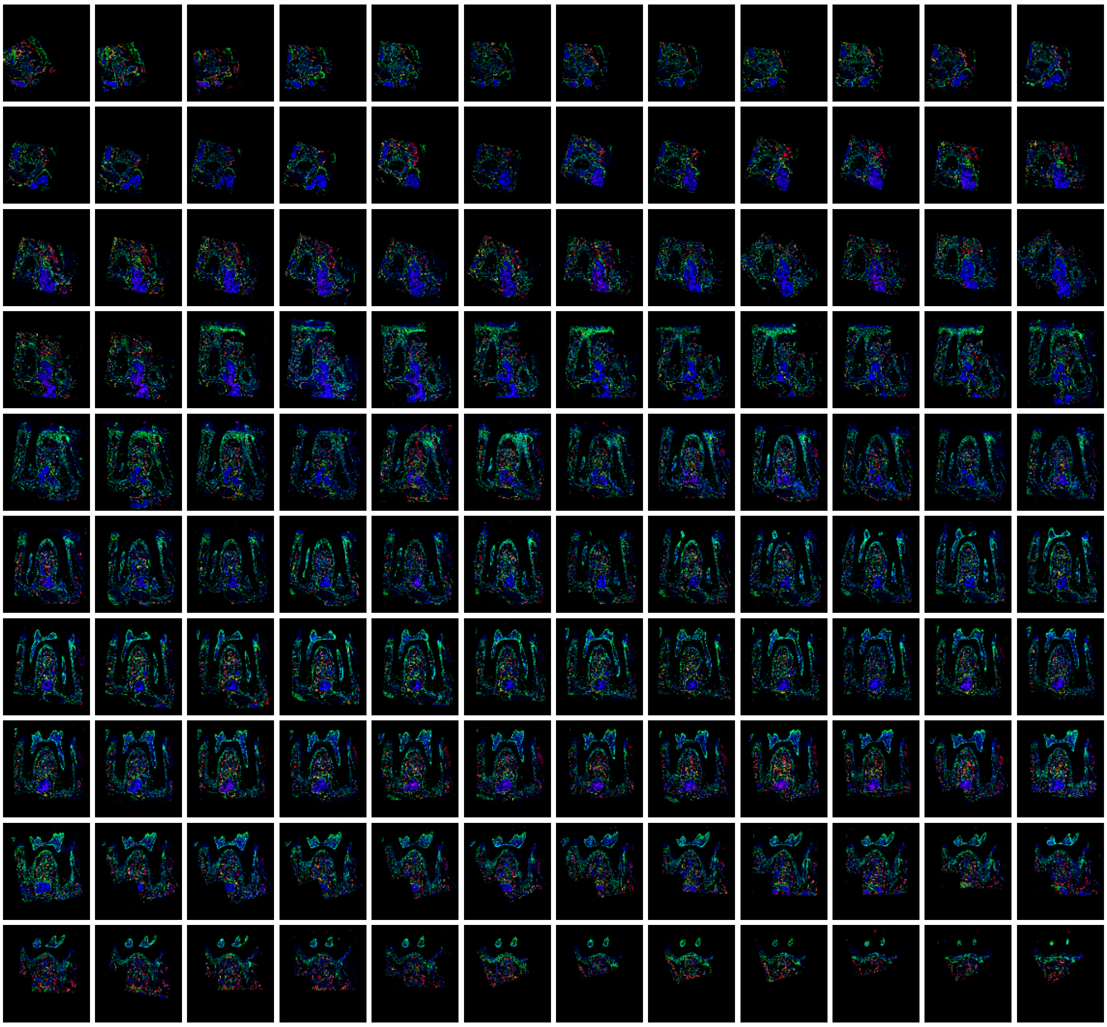

**Figure 7—figure supplement 1**. All consecutive slices (a total of 120 slices) for imaging of mandible M1 of *Pdgfra*$^{CreER}$ × *Nfatc1*$^{DreER}$ × LGRT mice (tracing 11 days). The images were acquired by confocal microscope, ZsGreen$^{+}$ cells in green, tdTomato$^{+}$ cells in red, DAPI in blue.

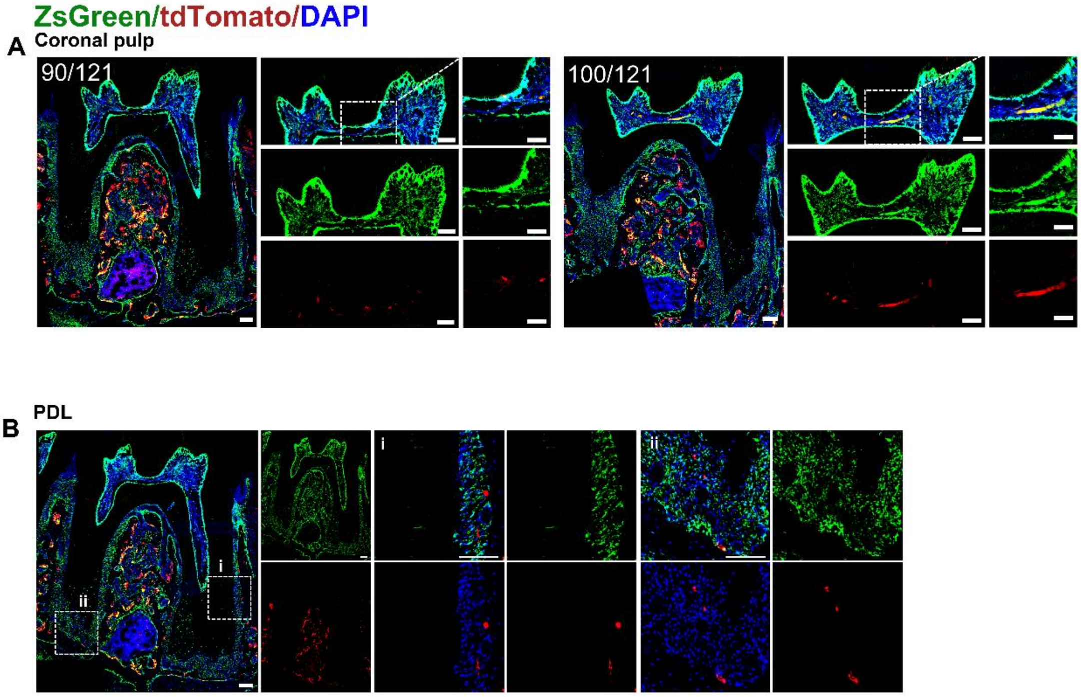


**Figure 7—figure supplement 2**. Representative images of coronal pulp (A) and PDL (B) acquired by confocal microscope of mandible M1 of *Pdgfrα*$^{CreER}$ × *Nfatc1*$^{DreER}$ × LGRT mice sample (tracing). Scale bar = 100 µm.

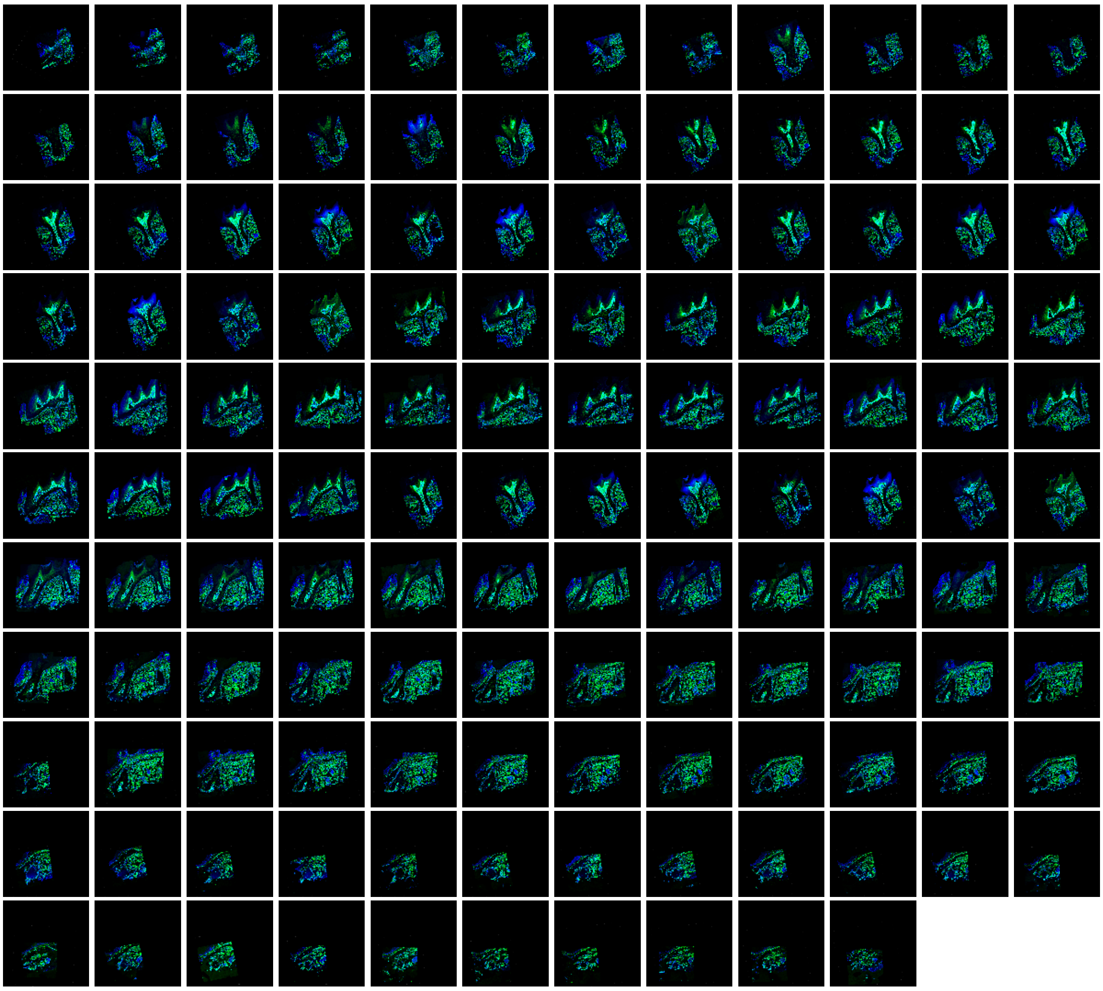

**Figure 8—figure supplement 1**. All consecutive slices (a total of 130 slices) for imaging of maxilla of *Pdgfra*$^{CreER}$ × IR1 mice. The images were acquired by confocal microscope, ZsGreen$^{+}$ cells in green and DAPI in blue.

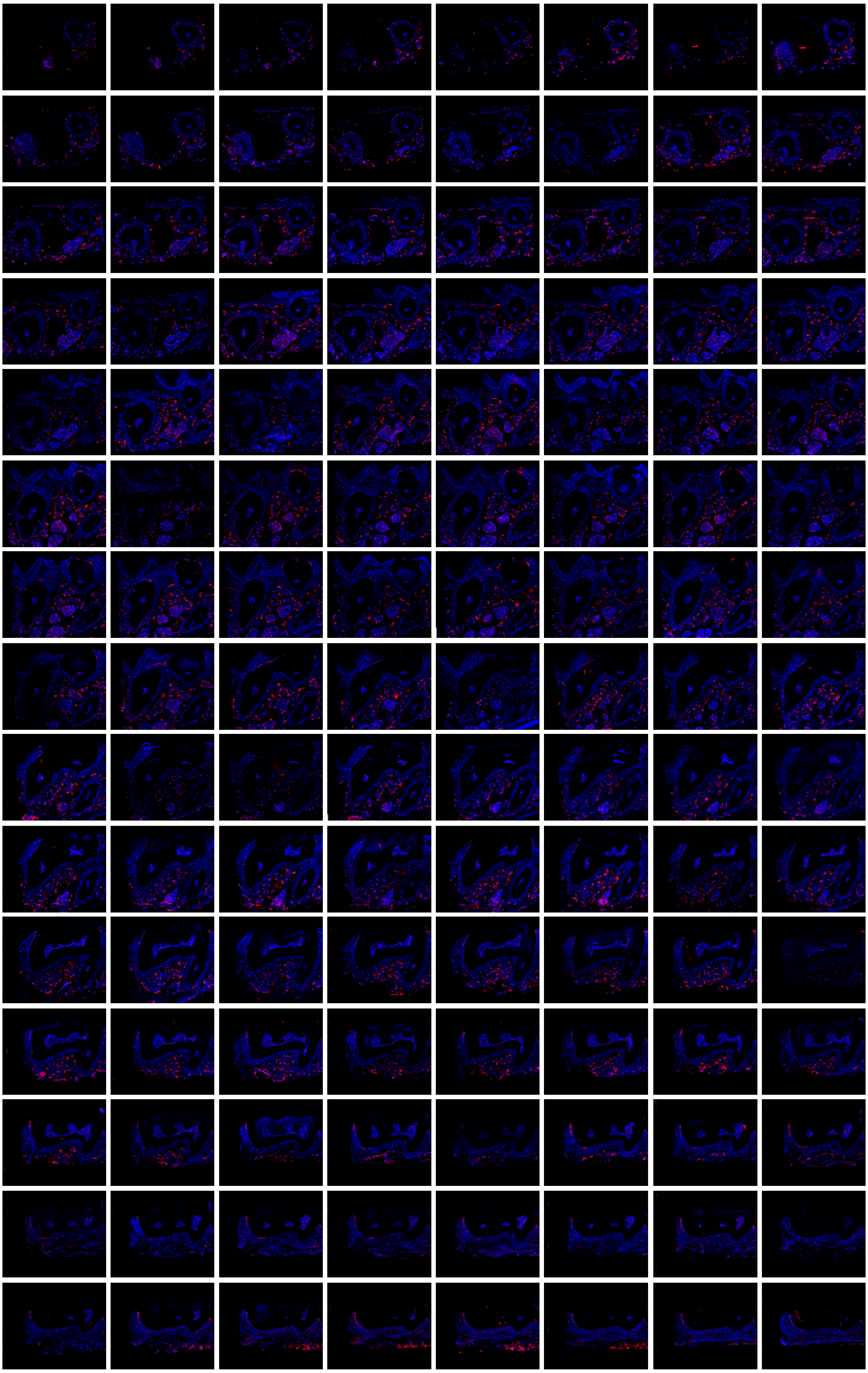

**Figure 8—figure supplement 2**. All consecutive slices (a total of 121 slices) for imaging of maxilla of *Nfatc1*$^{DreER}$ × IR1 mice. The images were acquired by confocal microscope, tdTomato$^{+}$ cells in red and DAPI in blue.

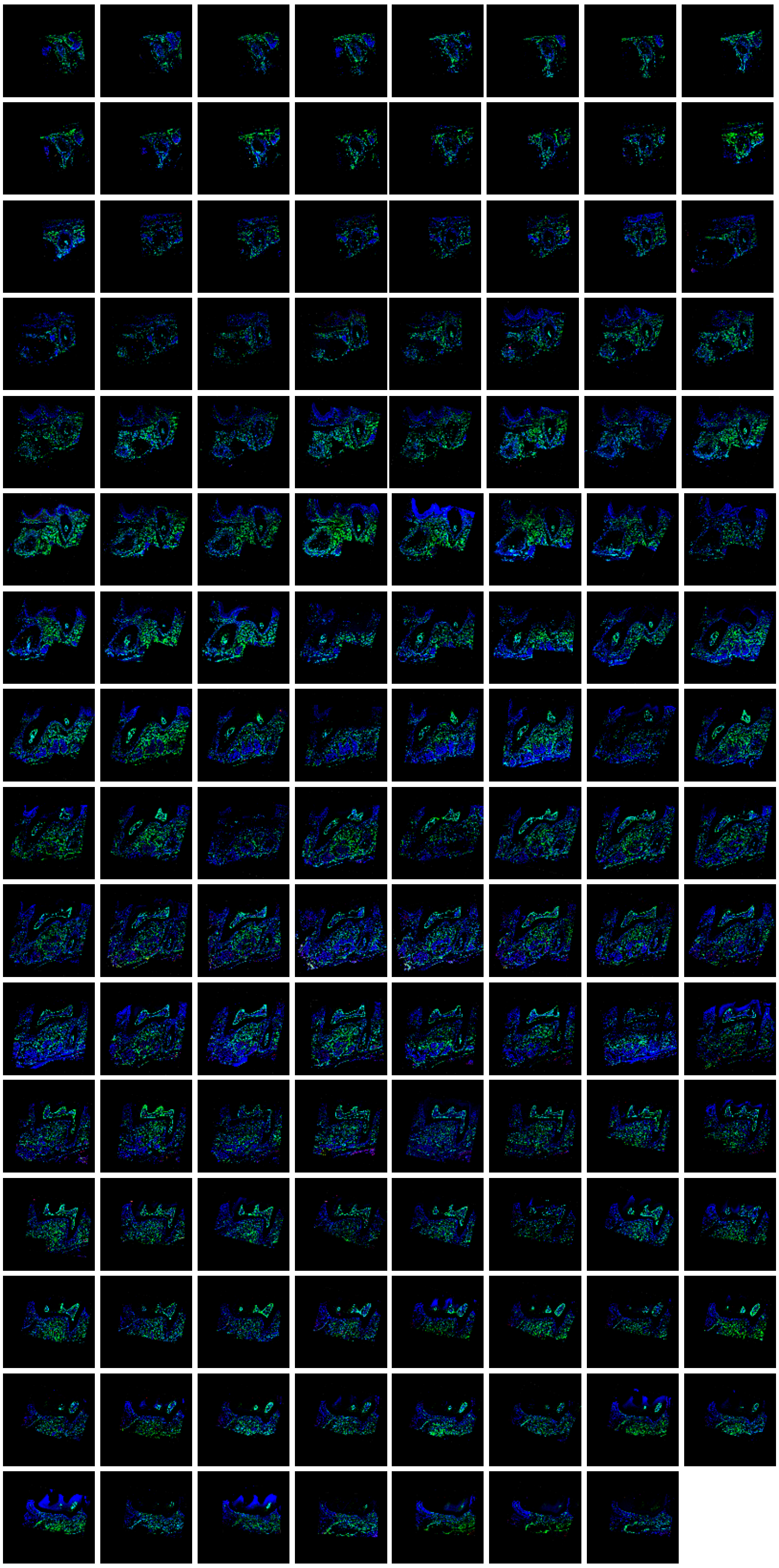

**Figure 8—figure supplement 3**. All consecutive slices (a total of 127 slices) for imaging of maxilla of $Pdgfra^{CreER}$ × $Nfatc1^{DreER}$ × IR1 mice sample. The images were acquired by confocal microscope, ZsGreen$^{+}$ cells in green, tdTomato$^{+}$ cells in red, DAPI in blue.

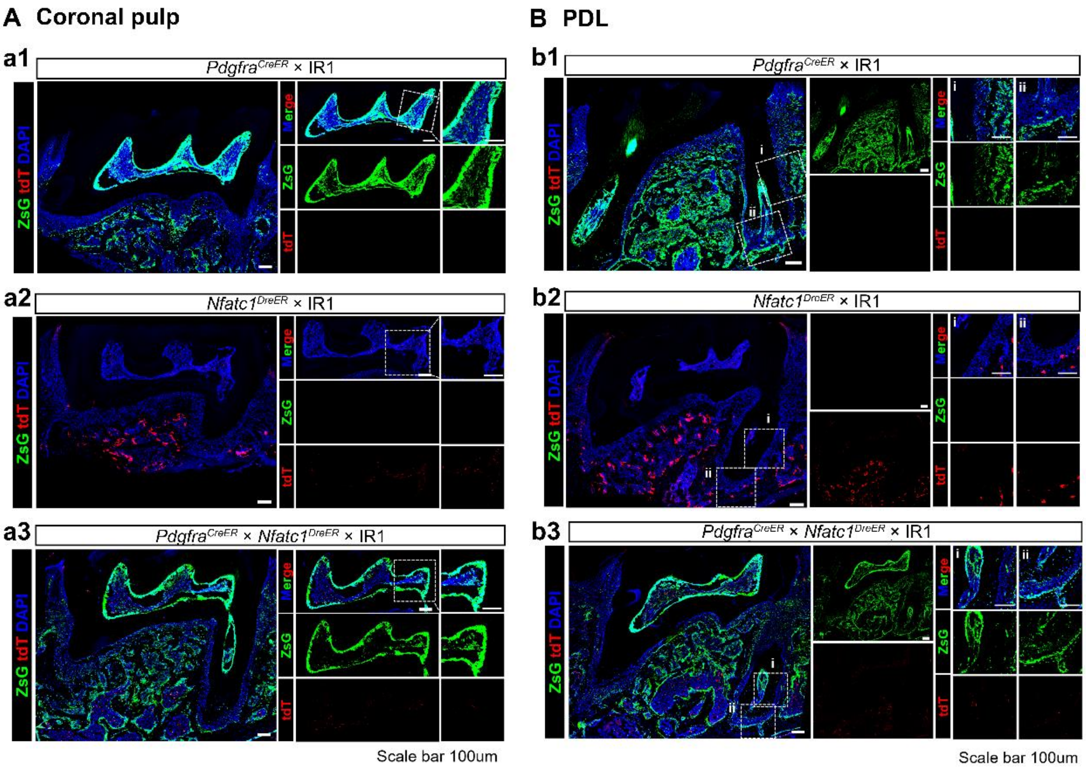


**Figure 8—figure supplement 4**. Representative images of coronal pulp (A) and PDL (B) acquired by confocal microscope of mandible M1 of *Pdgfra*$^{CreER}$ × IR1 (a1, b1), *Nfatc1*$^{DreER}$ × IR1 (a2, b2) and *Pdgfra*$^{CreER}$ × *Nfatc1*$^{DreER}$ × IR1 (a3, b3) mice. Scale bar = 100 μm.

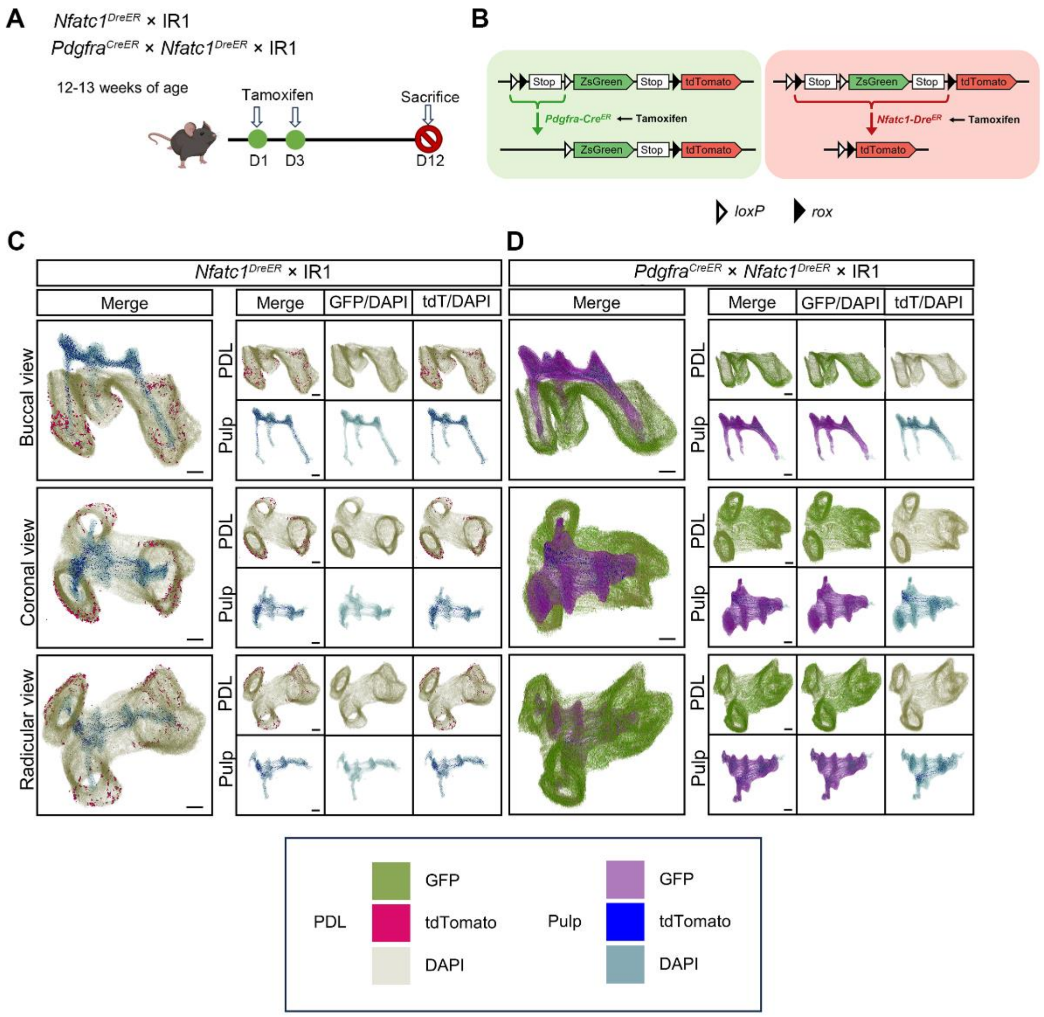


**Figure 8—figure supplement 5**. 3D reconstruction of maxilla M1 by DICOM-3D of maxilla M1 of *Nfatc1*$^{DreER}$ × IR1 and *Pdgfr-a*$^{CreER}$ × *Nfatc1*$^{DreER}$ × IR1 mice; in PDL, ZsGreen$^{+}$ cells in green, tdTomato$^{+}$ cells in rose red; in pulp, ZsGreen$^{+}$ cells in purple, tdTomato$^{+}$ cells in blue. The image stack was displayed in buccal view, coronal view, and radicular view. Scale bar: 200 μm.

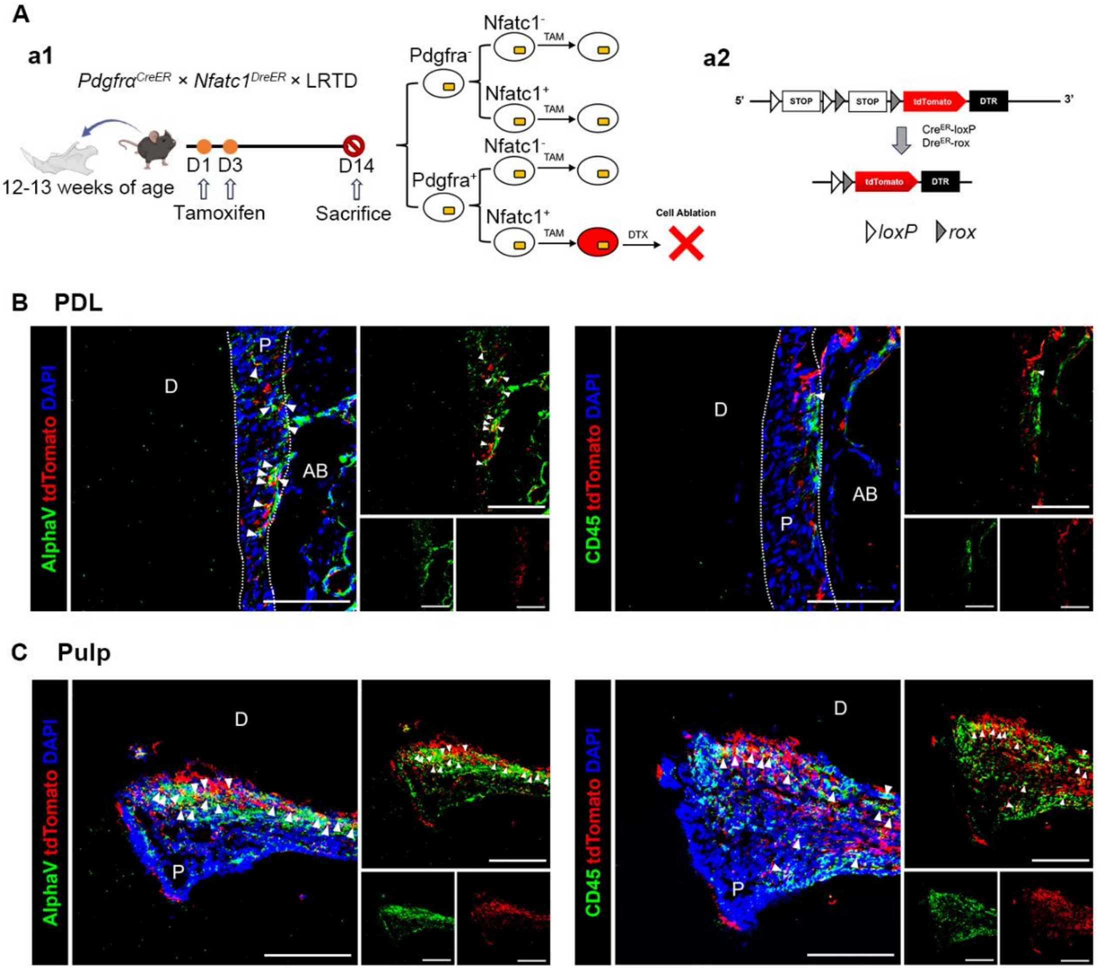


**Figure 9—figure supplement 1**. The identification of the types of PDGFR-α$^+$ & NFATc1$^+$ cells (red) of dental pulp and periodontal ligament tissues. (A) Schematic illustration of lineaging tracing in *Pdgfrα*$^{CreER}$ × *Nfatc1*$^{DreER}$ × LRTD mice. The mice were administrated with tamoxifen at D1 and D3, and sacrificed at D14. (B, C) Representative IF images of PDL B) and pulp C) of mandible M1 in P *Pdgfrα*$^{CreER}$ × *Nfatc1*$^{DreER}$ × LRTD mice showing the MSCs marker AlphaV (left) and hematopoietic marker CD45 (right). Arrows B, C) indicate the co-localization of AlphaV/CD45 and tdTomato. D: dentin; AB: alveolar bone. P: PDL B), pulp C). Scale bar: 100 μm.

**Legends of supplemental videos**

**Video 1**. Panoptic multicolor imaging of PDGFR-α$^+$ cells(green) & NFATc1$^+$ cells(red) in the pulp and PDL area of maxilla M1 of *Pdgfrα*$^{CreER}$ × *Nfatc1*$^{DreER}$ × LGRT mice (pulse), the whole-tissue imaging was achieved through TC procedure, related to Figure 2C.

**Video 2**. Panoptic multicolor imaging of PDGFR-α$^+$ cells & NFATc1$^+$ cells in the pulp and PDL area of maxilla M1 of *Pdgfrα*$^{CreER}$ × *Nfatc1*$^{DreER}$ × LGRT mice (pulse), the whole-tissue imaging was reconstructed from serial sections, related to Figure 3C. PDGFR-α$^+$ cells in green, NFATc1$^+$ cells in red and DAPI in blue

**Video 3**. Panoptic multicolor imaging of PDGFR-α$^+$ cells & NFATc1$^+$ cells in the pulp and PDL area of mandible M1 of *Pdgfra*$^{CreER}$ × *Nfatc1*$^{DreER}$ × LGRT mice (pulse), the whole-tissue imaging was reconstructed from serial sections, related to Figure 4C. PDGFR-α$^+$ cells in green, NFATc1$^+$ cells in red and DAPI in blue
**Video 4**. Panoptic multicolor imaging of ZsGreen$^+$ cells (green) & tdTomato$^+$ cells (red) in the pulp and PDL area of maxilla M1 of *Pdgfra*$^{CreER}$ × *Nfatc1*$^{DreER}$ × LGRT mice (tracing 11 days), the whole-tissue imaging was achieved through TC procedure, related to Figure 5B.
**Video 5**. Panoptic multicolor imaging of ZsGreen$^+$ cells & tdTomato$^+$ cells in the pulp and PDL area of maxilla M1 of *Pdgfra*$^{CreER}$ × *Nfatc1*$^{DreER}$ × LGRT mice (tracing 11 days), the whole-tissue imaging was reconstructed from serial sections, related to Figure 6C. ZsGreen$^+$ cells in green, tdTomato$^+$ cells in red and DAPI in blue
**Video 6**. Panoptic multicolor imaging of ZsGreen$^+$ cells (green) & tdTomato$^+$ cells (red) in the pulp and PDL area mandible M1 of *Pdgfra*$^{CreER}$ × *Nfatc1*$^{DreER}$ × LGRT mice (tracing 11 days), the whole-tissue imaging was reconstructed from serial sections, related to Figure 7C. ZsGreen$^+$ cells in green, tdTomato$^+$ cells in red and DAPI in blue
**Video 7**. Panoptic multicolor imaging of PDGFR-α$^+$ cells (green) & NFATc1$^+$ cells (red) in cranium of *Pdgfra*$^{CreER}$ × *Nfatc1*$^{DreER}$ × LGRT mice (pulse), the whole-tissue imaging was achieved through TC procedure.
**Video 8**. Panoptic multicolor imaging of PDGFR-α$^+$ cells (green) & NFATc1$^+$ cells (red) in cranial sagittal suture of *Pdgfra*$^{CreER}$ × *Nfatc1*$^{DreER}$ × LGRT mice (pulse), the whole-tissue imaging was achieved through TC procedure.
**Video 9**. Panoptic multicolor imaging of PDGFR-α$^+$ cells (green) & NFATc1$^+$ cells (red) in cranial coronal suture of *Pdgfra*$^{CreER}$ × *Nfatc1*$^{DreER}$ × LGRT mice (pulse), the whole-tissue imaging was achieved through TC procedure.
**Video 10**. Panoptic multicolor imaging of ZsGreen$^+$ cells in the pulp and PDL area of maxilla M1 of *Pdgfra*$^{CreER}$ × IR1 mice, the whole-tissue imaging was reconstructed from serial sections, related to Figure 8. ZsGreen$^+$ cells in green and DAPI in blue.
**Video 11**. Panoptic multicolor imaging of tdTomato$^+$ cells in the pulp and PDL area of maxilla M1 of *NFATc1*$^{DreER}$ × IR1 mice, the whole-tissue imaging was reconstructed from serial sections, related to Figure 8. tdTomato$^+$ cells in red and DAPI in blue.
**Video 12**. Panoptic multicolor imaging of ZsGreen$^+$ cells & tdTomato$^+$ cells in the pulp and PDL area of maxilla M1 of *Pdgfra*$^{CreER}$ × *NFATc1*$^{DreER}$ × IR1 mice, the whole-tissue imaging was reconstructed from serial sections, related to Figure 8. ZsGreen$^+$ cells in green, tdTomato$^+$ cells in red and DAPI in blue.
